# HDAC3 negatively regulates spatial memory in a mouse model of Alzheimer's disease

**DOI:** 10.1111/acel.12642

**Published:** 2017-08-03

**Authors:** Xiaolei Zhu, Sulei Wang, Linjie Yu, Jiali Jin, Xing Ye, Yi Liu, Yun Xu

**Affiliations:** ^1^ The State Key Laboratory of Pharmaceutical Biotechnology Department of Neurology Medical School Drum Tower Hospital Nanjing University Nanjing China; ^2^ Jiangsu Key Laboratory for Molecular Medicine Medical School of Nanjing University Nanjing China; ^3^ Nanjing Neuropsychiatry Clinic Medical Center Nanjing China; ^4^ Department of Neurology Nanjing Hospital of Traditional Chinese Medicine Nanjing China

**Keywords:** Alzheimer's disease, beta‐amyloid, histone deacetylase3, spatial memory

## Abstract

The accumulation and deposition of beta‐amyloid (Aβ) is a key neuropathological hallmark of Alzheimer's disease (AD). Histone deacetylases (HDACs) are promising therapeutic targets for the treatment of AD, while the specific HDAC isoforms associated with cognitive improvement are poorly understood. In this study, we investigate the role of HDAC3 in the pathogenesis of AD. Nuclear HDAC3 is significantly increased in the hippocampus of 6‐ and 9‐month‐old APPswe/PS1dE9 (APP/PS1) mice compared with that in age‐matched wild‐type C57BL/6 (B6) mice. Lentivirus ‐mediated inhibition or overexpression of HDAC3 was used in the hippocampus of APP/PS1 mice to investigate the role of HDAC3 in spatial memory, amyloid burden, dendritic spine density, glial activation and tau phosphorylation. Inhibition of HDAC3 in the hippocampus attenuates spatial memory deficits, as indicated in the Morris water maze test, and decreases amyloid plaque load and Aβ levels in the brains of APP/PS1 mice. Dendritic spine density is increased, while microglial activation is alleviated after HDAC3 inhibition in the hippocampus of 9‐month‐old APP/PS1 mice. Furthermore, HDAC3 overexpression in the hippocampus increases Aβ levels, activates microglia, and decreases dendritic spine density in 6‐month‐old APP/PS1 mice. In conclusion, our results indicate that HDAC3 negatively regulates spatial memory in APP/PS1 mice and HDAC3 inhibition might represent a potential therapy for the treatment of AD.

## Introduction

Alzheimer's disease (AD), the most common cause of dementia among the elderly, is a progressive neurodegenerative disease that affects 5.3 million people in the United States (Alzheimer's's, 2015). Although the precise mechanisms of AD are largely unknown, the deposition of beta‐amyloid (Aβ) is a key neuropathological hallmark. AD is closely associated with synaptic dysfunction, glial reactivity, and neuronal loss (Selkoe & Hardy, 2016). Aβ is derived from the proteolytic metabolism of transmembrane β‐amyloid precursor protein (APP). In the amyloidogenic pathway, Aβ is produced by sequential cleavage of APP by β‐secretase and γ‐secretase; in the nonamyloidogenic pathway, APP is consecutively cleaved by α‐secretase and γ‐secretase, which yields smaller Aβ fragments and neuroprotective sAPPα (Selkoe, 2013). In addition, Aβ clearance by enzyme‐mediated transport and degrading contributes to the dynamic balance of Aβ in the brain (Miners *et al*., 2011).

Histone acetylation, which is modulated by the activity of histone acetyltransferase (HAT) and histone deacetylase (HDAC), plays an important role in the pathogenesis of AD by altering chromatin structure and accessibility (Peixoto & Abel, 2013). The level of histone acetylation is significantly lower in both AD transgenic mice and postmortem human brains (Francis *et al*., 2009; Narayan *et al*., 2015). Compelling evidence has shown that targeting histone acetylation with HDAC inhibitors may improve synaptic plasticity and memory functions in AD models (Kilgore *et al*., 2010; Benito *et al*., 2015; Rumbaugh *et al*., 2015). Although some HDAC inhibitors are approved for the treatment of tumors by the Food and Drug Administration of the United States, the side effects of HDAC pan‐inhibitors limit their use (Duvic & Dimopoulos, 2016). Thus, the identification of a HDAC isoform specifically for the improvement of memory deficits would be of great value.

Histone deacetylase 3 is found in both the nucleus and the cytoplasm and is able to translocate between them, while other class I HDACs are mainly located in the nucleus (Yang *et al*., 2002). Additionally, HDAC3 is widely expressed in the brain and is considered neurotoxic. Overexpression of HDAC3 induces the death of cerebellar granule neurons, while inhibition of HDAC3 protects against the effects of low potassium (Bardai & D'Mello, 2011). Inhibition of HDAC3 significantly enhances long‐term memory in object recognition tasks (McQuown *et al*., 2011). HDAC3 also negatively regulates cocaine‐induced conditioned place preference acquisition (Malvaez *et al*., 2013). RGFP966, a selective HDAC3 inhibitor, has been shown to affect sensory cortical plasticity and memory formation (Bieszczad *et al*., 2015). However, whether HDAC3 contributes to the development of AD is still debatable (Graff *et al*., 2012; Rumbaugh *et al*., 2015; Krishna *et al*., 2016). In this study, we investigated whether HDAC3 modulates spatial memory and pathogenesis in an AD mouse model.

## Results

### HDAC3 is increased in the hippocampal nuclei of APPswe/PS1dE9 (APP/PS1) mice

**Figure 1:**
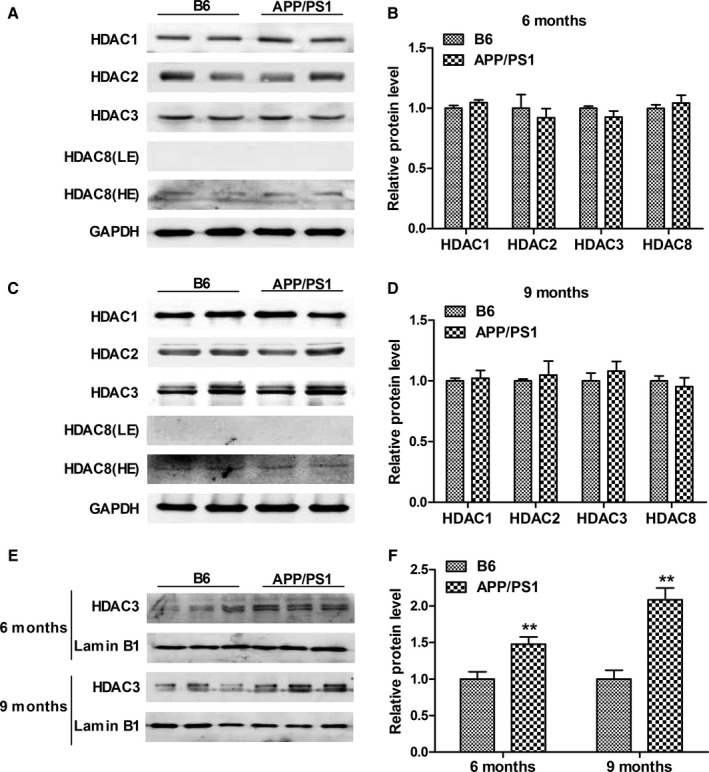
Nuclear histone deacetylase 3 (HDAC3) is increased in the hippocampus of 6‐ and 9‐month‐old APPswe/PS1dE9 (APP/PS1) mice. (A) The levels of Class I HDACs (HDAC1, HDAC2, HDAC3, and HDAC8) in the hippocampus of 6‐month‐old APP/PS1 mice were determined by Western blot. LE: low exposure; HE: high exposure. *n *=* *4–6 mice per group. (B) Graph represents quantification of the signal intensities normalized to GAPDH as a loading control. (C) The levels of HDAC1, HDAC2, HDAC3, and HDAC8 in the hippocampus of 9‐month‐old APP/PS1 mice were determined by Western blot. *n *=* *4–6 mice per group. (D) Graph represents quantification of the signal intensities normalized to GAPDH as a loading control. (E) The level of nuclear HDAC3 was determined by Western blot. *n *=* *6–8 mice per group. (F) Graph represents quantification of the signal intensities normalized to lamin B1 as a loading control. **P <* *0.01.

Given that class I HDACs play critical roles in synaptic function and memory formation, we first determined the levels of HDAC1, HDAC2, HDAC3, and HDAC8 in the hippocampus of APP/PS1 mice. As shown in Fig. 1A–D, the protein levels of HDAC1, HDAC2, HDAC3, and HDAC8 were not altered in the hippocampus of 6‐ and 9‐month‐old APP/PS1 mice (*P *>* *0.05). The expression of HDAC8 appeared much lower than that of the other class I HDACs, which was consistent with previous studies (Broide *et al*., 2007; Anderson *et al*., 2015). In addition, the mRNA level of HDAC3 was not significantly changed in the hippocampus of 6‐ and 9‐month‐old APP/PS1 mice (*P *>* *0.05; Fig. S1). The subcellular distribution of HDAC3 was examined next as, unlike other class I HDACs mainly located in the nucleus, HDAC3 has been shown to shuttle between the nucleus and the cytoplasm (Yang *et al*., 2002). The protein level of HDAC3 was significantly increased in the hippocampal nuclei of 6‐ and 9‐month‐old APP/PS1 mice (*P *=* *0.008 and *P *<* *0.001, respectively; Fig. 1E,F), which indicated that the increase in HDAC3 in the nuclei might be associated with the pathogenesis of AD.

### Inhibition of HDAC3 attenuates the spatial memory deficits of APP/PS1 mice

**Figure 2:**
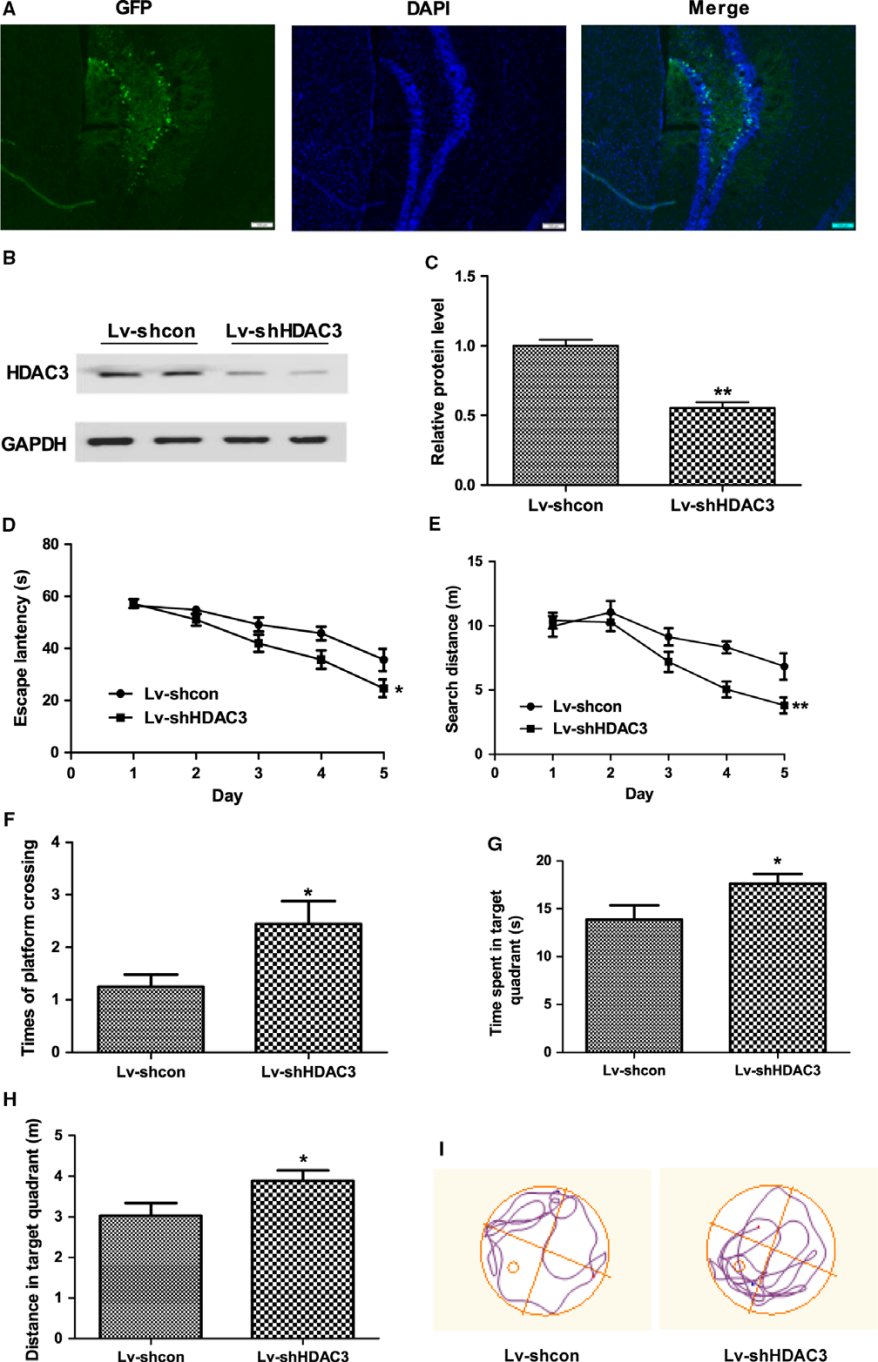
Lentivirus‐mediated inhibition of histone deacetylase 3 (HDAC3) in the hippocampus attenuates spatial memory deficits in 9‐month‐old APPswe/PS1dE9 (APP/PS1) mice. Nine‐month‐old APP/PS1 mice were injected with lenti‐shHDAC3 or lenti‐shcon, and the Morris water maze (MWM) tests were performed 30 days later. (A) Representative image of lenti‐shHDAC3‐injected hippocampus of 9‐month‐old APP/PS1 mice. (scale bar = 100 μm). (B) The level of HDAC3 in the hippocampus after lenti‐shHDAC3 injection was determined by Western blot. *n *=* *4 mice per group. (C) Graph represents quantification of the signal intensities normalized to GAPDH as a loading control. In the acquisition trial, the escape latency (D) and searching distance (E) were analyzed in lenti‐shHDAC3 (*n *=* *14)‐ and lenti‐shcon (*n *=* *12)‐injected mice, and the number of target platform crossings (F), time in the target quadrant (G), and swimming distance in the target quadrant (H) were recorded in the probe trials. (I) A representative image of path tracings in the probe test on day 6. *P <* *0.05, **P <* *0.01.

To determine the therapeutic potential of HDAC3, lentivirus‐mediated inhibition of HDAC3 was used in the hippocampus of APP/PS1 mice (9 months old). As shown in Fig. 2A, GFP‐tagged lentivirus was mainly detected in the hippocampus. The efficiency of lenti‐shHDAC3 was confirmed using Western blot (*P *<* *0.001; Fig. 2B,C). To investigate the effect of HDAC3 inhibition on the spatial memory of APP/PS1 mice, the Morris water maze (MWM) test was performed 30 days after the lentivirus injection. As shown in Fig. 2D, compared with lenti‐shcon‐injected APP/PS1 mice, lenti‐shHDAC3‐injected APP/PS1 mice showed reduced escape latency during training (groups: *F*(1, 96) = 7.510, *P *=* *0.013; days: *F*(4, 96) = 44.422, *P *<* *0.001; group × day: *F*(4, 96) = 0.915, *P *=* *0.063). The searching distance of the APP/PS1 group was also decreased following HDAC3 inhibition (groups: *F*(1, 96) = 8.343, *P *=* *0.009; days: *F*(4, 96) = 28.697, *P *<* *0.001; group x day: *F*(4, 96) = 4.115, *P *=* *0.055; Fig. 2E). During the probe trial, the time of target platform crossings of lenti‐shHDAC3‐injected APP/PS1 mice was increased (*P *=* *0.021; Fig. 2F). Moreover, the time spent and swimming distance in the target quadrant were significantly increased in lenti‐shHDAC3‐injected APP/PS1 mice (*P *=* *0.037 and *P *=* *0.039, respectively; Fig. 2G–I).

To further investigate the role of HDAC3 in the pathogenesis of AD, a lentivirus overexpressing HDAC3 was injected into the hippocampus of APP/PS1 mice (6 months old), and the results of Western blot confirmed the overexpression of HDAC3 (Fig. S2A, Supporting information). HDAC3 overexpression significantly increased the latency (groups: *F*(1, 48) = 15.583, *P *=* *0.003; days: *F*(4, 48) = 29.879, *P *<* *0.001; group x day: *F*(4, 48) = 0.915, *P *=* *0.516; Fig. S2B, Supporting information) and searching distance (groups: *F*(1, 48) = 8.038, *P *=* *0.020; days: *F*(4, 48) = 13.924, *P *<* *0.001; group × day: *F*(4, 48) = 1.791, *P *=* *0.152; Fig. S2C, Supporting information) of APP/PS1 mice in the acquisition trial and moderately decreased the time of target platform crossings and time spent in the target quadrant in the probe trial (*P *=* *0.586 and *P *=* *0.664, respectively; Fig. S2D,E Supporting information). Thus, these results showed that HDAC3 was a negative regulator of spatial memory in APP/PS1 mice.

### Inhibition of HDAC3 decreased Aβ levels in the brains of APP/PS1 mice

**Figure 3:**
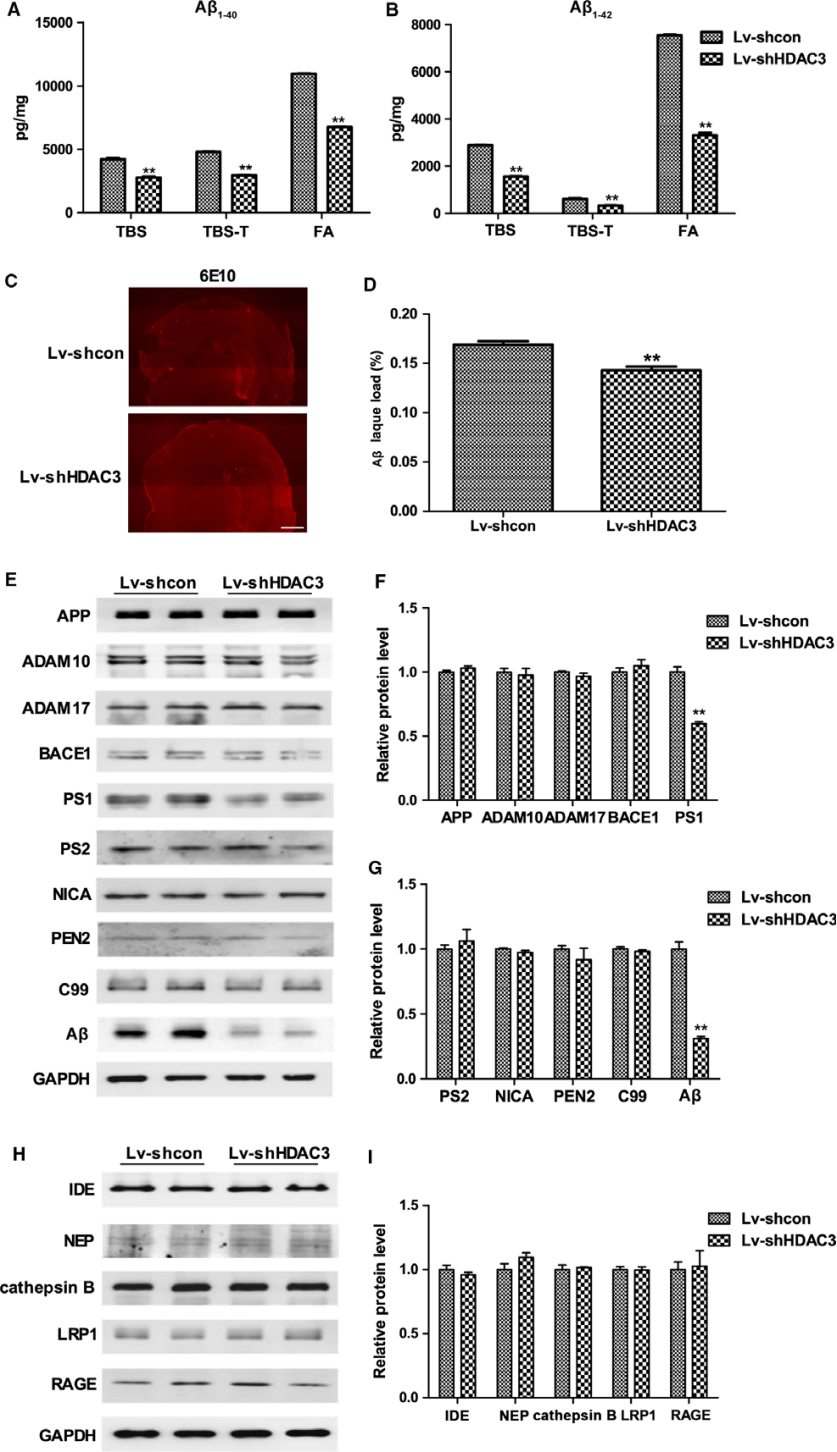
Inhibition of histone deacetylase 3 (HDAC3) decreases Aβ levels in the hippocampus of 9‐month‐old APPswe/PS1dE9 (APP/PS1) mice. (A) The levels of TBS‐, TBS‐X‐, and FA‐soluble Aβ_1‐40_ were determined by ELISA in the hippocampus. *n *=* *4–6 mice per group. (B) The levels of TBS‐, TBS‐X‐, and FA‐soluble Aβ_1–42_ were determined by ELISA in the hippocampus. *n *=* *4–6 mice per group. (C) Representative image of Aβ staining (6E10) in the brains of lenti‐shHDAC3‐injected APP/PS1 mice. (scale bar = 250 μm). *n *=* *4 mice per group. (D) The area percentage of 6E10‐positive Aβ plaque load in the brains. (E) The levels of APP and secretases in the hippocampus of lenti‐shHDAC3‐injected APP/PS1 mice were examined by Western blot. *n *=* *4–5 mice per group. (F, G) Graph represents quantification of the signal intensities normalized to GAPDH as a loading control. (H) The levels of Aβ‐metabolism‐associated enzymes were determined in the hippocampus of lenti‐shHDAC3‐injected APP/PS1 mice. *n *=* *4–5 mice per group. (I) Graph represents quantification of the signal intensities normalized to GAPDH as a loading control. **P <* *0.01.

To determine whether the memory improvement induced by HDAC3 inhibition was associated with Aβ levels, the levels of Aβ_1–40_ and Aβ_1–42_ in the hippocampus of APP/PS1 mice after the injection of lentivirus were examined by ELISA. In the TBS fractions (soluble Aβ), inhibition of HDAC3 significantly decreased the level of Aβ_1–40_ and Aβ_1–42_ in the hippocampus (*P *<* *0.001; Fig. 3A,B). In addition, the levels of Aβ_1–40_ and Aβ_1–42_ in TBS‐X and FA fractions (plaque‐associated Aβ) were decreased in the hippocampus of APP/PS1 mice (*P *<* *0.001; Fig. 3A,B), which indicated that the inhibition of HDAC3 could decrease both soluble and insoluble Aβ levels. Next, we investigated whether HDAC3 inhibition regulated the deposition of amyloid plaques using IFA. As shown in Fig. 3C,D, inhibition of HDAC3 significantly reduced amyloid plaques areas in the brains of APP/PS1 mice (*P *=* *0.001). These results indicated that HDAC3 inhibition decreased the Aβ_1–40_ and Aβ_1–42_ levels and amyloid plaques in APP/PS1 mice.

The impact of HDAC3 overexpression on the Aβ levels and amyloid plaques in the brains of APP/PS1 mice was also examined. As shown in Fig. S3A,B (Supporting information), the levels of TBS‐, TBS‐X‐ and FA‐soluble Aβ_1–40_ and Aβ_1–42_ in the hippocampus were markedly increased in lenti‐HDAC3‐injected APP/PS1 mice. In addition, the areas of amyloid plaques were increased after HDAC3 overexpression (*P *=* *0.014; Fig. S3C,D Supporting information).

### HDAC3 regulates Aβ generation in the hippocampus of APP/PS1 mice

Next, we explored whether HDAC3 modulated the generation and clearance of Aβ in the hippocampus of APP/PS1 mice. The levels of APP, α‐secretase (ADAM10/ADAM17), β‐secretase (BACE1), and γ‐secretase (PS1/PS2/PEN2/Nicastrin) were determined by Western blot. The levels of PS1 and Aβ were significantly reduced in the hippocampus of lenti‐shHDAC3‐injected APP/PS1 mice (*P *<* *0.001; Fig. 3E–G), while the other secretases were not significantly changed (*P *>* *0.05; Fig. 3E–G). In addition, the levels of Aβ‐metabolism‐associated enzymes, including IDE, NEP, cathepsin B, LRP1, and RAGE, were not significantly affected following HDAC3 inhibition (*P *>* *0.05; Fig. 3H,I), suggesting that HDAC3 inhibition might decrease the expression of PS1 and ameliorate the amyloid burden in the hippocampus of APP/PS1 mice. Interestingly, overexpression of HDAC3 in the hippocampus significantly upregulated the level of PS1 and downregulated the level of ADAM10 (*P *<* *0.001; Fig. S4A–C, Supporting information) and did not affect the expression levels of Aβ‐metabolism‐associated enzymes (*P > *0.05; Fig. S4D,E, Supporting information). Collectively, HDAC3 might modulate the expression of PS1 to affect APP processing, leading to an imbalanced Aβ burden.

### Inhibition of HDAC3 increases dendritic spine density in the hippocampus of APP/PS1 mice

**Figure 4:**
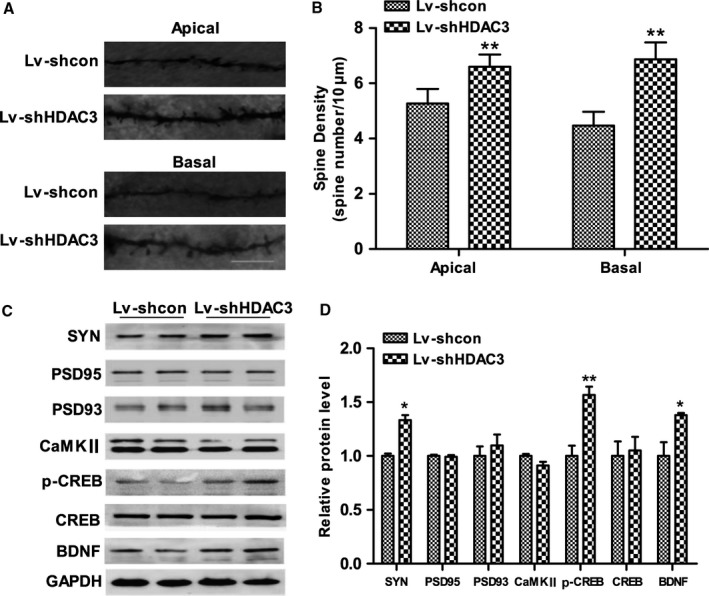
Inhibition of histone deacetylase 3 (HDAC3) increases dendritic spine density in the hippocampus of APPswe/PS1dE9 (APP/PS1) mice. (A) Representative image of the Golgi‐stained apical and basal dendrites in CA1 pyramidal neurons. (scale bar = 10 μm). *n *=* *3–4 mice per group. (B) Graph represents quantification of the apical and basal dendritic spine density. (C) The levels of synaptophysin, PSD95, PSD93, CaMKII, p‐CREB, CREB, and BDNF in the hippocampus of lenti‐shHDAC3‐injected APP/PS1 mice were determined by Western blot. *n *=* *4–5 mice per group. (D) Graph represents quantification of the signal intensities normalized to GAPDH as a loading control. *P <* *0.05, **P <* *0.01.

Dendritic spine density is reduced in soluble Aβ‐induced neuronal cells and AD mouse models and is associated with synaptic loss and cognitive dysfunction. In this study, we examined the spine density in CA1 pyramidal neurons in the hippocampus of APP/PS1 mice using Golgi staining. As shown in Fig. 4A,B, the spine density of apical and basal dendrites was significantly increased in the hippocampus of Lv‐shHDAC3‐injected APP/PS1 mice (*P *=* *0.009 and *P *=* *0.005, respectively). In addition, we examined the expression of some key proteins correlated with synaptic function. The level of synaptophysin, a presynaptic vesicle protein, was significantly increased (*P *<* *0.001; Fig. 4C,D), while the levels of PSD95, PSD93, and CaMKII, three postsynaptic proteins, were not changed in the hippocampus of Lv‐shHDAC3‐injected APP/PS1 mice (*P *>* *0.05; Fig. 4C,D). HDAC3 inhibition also resulted in a significant increase in p‐CREB and BDNF in the hippocampus of APP/PS1 mice (*P *=* *0.001 and *P *=* *0.031, respectively; Fig. 4C,D), which played critical roles in neurite outgrowth, synaptic plasticity and memory formation. Similarly, HDAC3 overexpression decreased the spine density of apical and basal dendrites in the hippocampus of APP/PS1 mice (*P *=* *0.002 and *P *=* *0.004; Fig. S5A,B, Supporting information). The expression levels of synaptophysin and BDNF were also reduced in the hippocampus of Lv‐HDAC3‐injected APP/PS1 mice (*P *<* *0.001; Fig. S5C,D, Supporting information). Overall, these data indicated that HDAC3 might regulate the expression levels of synaptophysin and BDNF and modulate dendritic spine density.

### HDAC3 regulates microglial activation in the hippocampus of APP/PS1 mice

**Figure 5:**
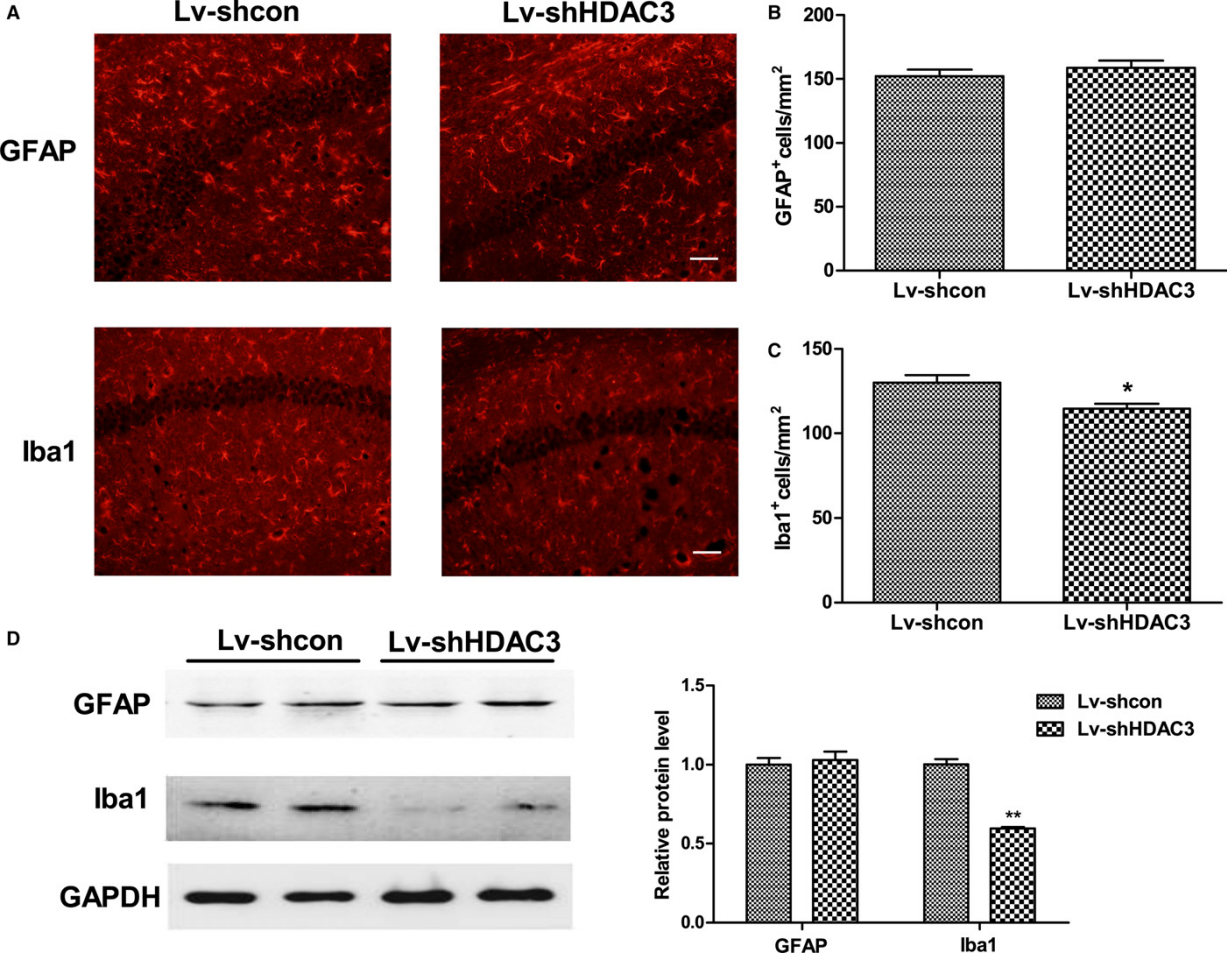
Inhibition of HDAC3 attenuates microglial activation in the hippocampus of APP/PS1 mice. (A) Representative image of astrocytes and microglia stained with GFAP and Iba‐1, respectively, in the hippocampus of lenti‐shHDAC3‐injected APP/PS1 mice. (scale bar = 50 μm). *n *=* *4–5 mice per group. (B, C) Graph represents quantification of the signal intensities. (D) The levels of GFAP and Iba‐1 in the hippocampus were determined by Western blot. *n *=* *4–6 mice per group. (E) Graph represents quantification of the signal intensities normalized to GAPDH as a loading control. *P <* *0.05, **P <* *0.01.

To determine the effect of HDAC3 on neuroinflammation, the expression levels of Iba‐1 (a marker of microglia) and GFAP (a marker of astrocyte) were measured using IFA and Western blot. HDAC3 inhibition attenuated the activation of microglia in the hippocampus of APP/PS1 mice (*P *=* *0.018; Fig. 5A–C). The results of Western blot confirmed these data (*P *<* *0.001; Fig. 5D,E). The level of GFAP was not affected following HDAC3 inhibition or HDAC3 overexpression as demonstrated by IFA and Western blot (Fig. 5 and Fig. S6, Supporting information). Furthermore, the level of Iba‐1 was significantly increased in the hippocampus of Lv‐HDAC3‐injected APP/PS1 mice as demonstrated by IFA and Western blot (*P *=* *0.039 and *P *=* *0.032, respectively; Fig. S6, Supporting information). In conclusion, HDAC3 negatively regulated the activation of microglia in the hippocampus of APP/PS1 mice.

## Discussion

In the current study, we investigate the possible effect of HDAC3 on spatial memory deficits and the pathogenesis of AD, and our data have shown that (i) nuclear HDAC3 is significantly increased in the hippocampus of 6‐ and 9‐month‐old APP/PS1 mice compared with that in age‐matched wild‐type C57BL/6 (B6) mice; (ii) lentivirus‐mediated inhibition of HDAC3 in the hippocampus attenuates spatial memory deficits, decreases amyloid plaque load and Aβ levels, alleviates microglial activation, and increases dendritic spine density in 9‐month‐old APP/PS1 mice; and (iii) lentivirus‐mediated overexpression of HDAC3 in the hippocampus increases Aβ levels, activates microglia, and decreases dendritic spine density in 6‐month‐old APP/PS1 mice. Furthermore, inhibition or overexpression of HDAC3 does not alter the levels of phosphorylated tau in the hippocampus of 6‐ and 9‐month‐old APP/PS1 mice (Fig. S7, Supporting information).

Histone deacetylase 3 is the only class I HDAC located in both the nucleus and the cytoplasm and is also found in the plasma membrane (Yang *et al*., 2002). In the nucleus, HDAC3 interacts with the nuclear receptor corepressor and the silencing mediator of retinoic and thyroid receptors to regulate transcription and chromatin structure (You *et al*., 2013). The role of HDAC3 in the cytoplasm is less studied and might be associated with protein retention and signaling transduction. HDAC3 is cleaved by the caspase‐dependent pathway and translocated to the cytoplasm during apoptosis, while nuclear accumulation of HDAC3 can inhibit the progression of apoptosis (Escaffit *et al*., 2007). Increased nuclear, not cytoplasmic HDAC3 has been found in pancreatic cancer tissues and correlated with an advanced clinical stage and worse prognoses (Jiao *et al*., 2016). However, whether the subcellular translocation of HDAC3 contributes to the pathogenesis of AD is unknown and needs further study.

According to the molecular brake pad hypothesis (McQuown & Wood, 2011), HDACs act as molecular brake pads by repressing the transcription of genes required for memory formation, and the inhibition of specific HDAC isoforms will increase chromatin accessibility to facilitate transcription‐dependent memory processes. The role of HDAC3 in the pathogenesis of AD is controversial. The expression of HDAC3 is not altered in *postmortem* AD brains compared with that in healthy controls, as demonstrated by immunohistochemistry (Graff *et al*., 2012). HDAC3 inhibition by intraperitoneally injected (IP injected) RGFP966 exerts no effect on synaptogenesis and memory function, while RGFP963, an inhibitor of HDAC1, HDAC2, and HDAC3, induces synaptogenesis and rescues memory dysfunction in 6‐month‐old APP/PS1 mice, as demonstrated by the contextual fear conditioning test (Rumbaugh *et al*., 2015). However, RGFP966 attenuates the impaired long‐term potential (LTP) induced by Aβ_1–42_ oligomer using whole‐cell voltage‐clamp and field recording techniques (Krishna *et al*., 2016). Here, our data provide the first evidence that the inhibition of HDAC3 by lentivirus‐mediated shRNA in the hippocampus attenuates spatial memory deficits and decreases amyloid plaque load and Aβ levels in 9‐month‐old APP/PS1 mice. We speculate that at least the three following reasons contribute to the inconsistence of these studies: (i) lentivirus‐mediated inhibition of HDAC3 is used in this study, which inhibits not only deacetylase activity but also the expression of HDAC3 (considering that HDAC3 also has several deacetylase‐independent functions (Sun *et al*., 2013; Adikesavan *et al*., 2014), inhibition of the expression using a genetic knockout or knockdown approach might be more reasonable); (ii) RGFP966 is IP injected for 12 days at a dose of 30 mg kg^−1^ as previously described, but the time‐dependent and dose‐dependent effects of RGFP966 on the memory function of APP/PS1 mice are not shown; and (iii) although contextual fear conditioning and MWM tests are generally considered hippocampus‐dependent tasks, there are different brain regions involved, which might lead to inconsistent results (Puzzo *et al*., 2014). Interestingly, inhibition of HDAC3 activity by RGFP966 augments LTP and re‐establishes synaptic tagging and capture in aged hippocampal CA1 neurons (Sharma *et al*., 2015), which indicated that HDAC3 might contribute to both normal aging and AD pathogenesis.

Histone deacetylase inhibition has been shown to alter the dynamic of Aβ production and clearance. TSA increases the levels of Aβ_1–40_ and Aβ_1–42_ in the plasma of 9‐month‐old APP/PS1 mice, which indicates that it might facilitate the clearance of Aβ from the brain (Yang *et al*., 2014). Valproic acid (VPA), another pan‐HDAC inhibitor, reduces Aβ levels and inhibits plaque formation in APP23 and APP23/PS45 mice (Qing *et al*., 2008). Our results also demonstrate that inhibition of HDAC3 in the hippocampus of APP/PS1 mice decreases Aβ levels and alleviates amyloid plaque formation, which might be associated with decreased PS‐1 levels. An autosomal dominant mutation of PS‐1/PS‐2 leads to the early onset of familial Alzheimer's disease (FAD), and the PS1 complex displays greater γ‐secretase activity than the PS2 complex (Lai *et al*., 2003), which indicates that PS1 might be a critical catalytic component of γ‐secretase. Therefore, further studies are warranted to investigate the possible role of PS1 in HDAC3 inhibition‐mediated neuroprotective effects.

Previous studies have demonstrated that synaptic dysfunction appears in the early stages of AD, and different Aβ species, including amyloid plaques and intracellular Aβ, are associated with the synapse loss (Herms & Dorostkar, 2016). Here, we have shown that inhibition of HDAC3 regulates the synapse‐associated proteins synaptophysin, p‐CREB, and BDNF in the hippocampus of APP/PS1 mice and significantly increases spine density in CA1 pyramidal neurons. It was a limitation of our study that the particular types of dendritic spines and the correlation with the synaptic function were not analyzed. Previous studies have shown that RGFP966 ameliorates Aβ_1‐42_ oligomer‐induced LTP impairment in both single cell and a population of cells among the CA1 pyramidal neurons (Krishna *et al*., 2016), which is consistent with our results. However, systemic delivery of RGFP966 exerts no effect on spine density and the maturation of synaptic function (Rumbaugh *et al*., 2015). As HDAC3^−/−^ mice are embryonically lethal, conditional HDAC3 knockout mice might be used for further studies.

The activation of microglia and the release of proinflammatory factors also contribute to the pathogenesis of AD. The reactive microglia surround Aβ plaques in the brains of patients with AD, and Aβ induces the reactivation of microglia both *in vitro* and *in vivo* (Calsolaro & Edison, 2016). In addition, pharmacological inhibition of microglia attenuates contextual memory in AD mice without altering the amyloid levels (Spangenberg *et al*., 2016), which indicates that the protective effects might not be mediated by amyloid pathology. Recent studies have demonstrated that the R47H variant of triggering receptor expressed on myeloid cells 2 (TREM2), a receptor expressed in microglia, significantly increases the risk of nonfamilial AD (Guerreiro *et al*., 2013; Jonsson *et al*., 2013). In this study, our data demonstrate that HDAC3 inhibition in APP/PS1 mice results in a significant decrease in reactive microglia, while it does not alter astrocytosis. Furthermore, our recent data have shown that inhibition of HDAC3 by RGFP966 attenuates LPS‐induced primary microglia reactivation and Toll‐like receptor signaling pathway *in vitro*, which might be associated with the phosphorylation of STAT5 and STAT3 (Xia *et al*., 2017). Considering that inhibition of HDAC3 attenuated the amyloid pathology, whether the ameliorated microgliosis is directly regulated by HDAC3 inhibition requires further studies. Nonetheless, the anti‐inflammatory effect of HDAC3 inhibition on APP/PS1 mice indicates that it might be a potential target for AD treatment.

In conclusion, our data show that inhibition of HDAC3 ameliorates spatial memory impairment and improves AD‐related neuropathogenesis, while HDAC3 overexpression increases the amyloid burden in AD mice, which suggests that inhibition of HDAC3 is a potential therapy for the treatment of AD.

## Experimental Procedures

### Animals and treatments

APP/PS1 mice were obtained from the Model Animal Research Center of Nanjing University, and age‐matched B6 mice were used as controls. The delta exon 9 variant of PS1 specifically produces Aβ_1–42_, which is more prone to aggregation and leads to an earlier onset of amyloid plaques. APP/PS1 mice display visible amyloid plaques at 5–6 months of age, and memory deficits are detected at later stages (Jankowsky *et al*., 2004). Male and female APP/PS1 mice with similar numbers were used in this study. The lentivirus that overexpressed or inhibited HDAC3 was purchased from Shanghai Genchem. The HDAC3‐RNAi lentivirus (lenti‐shHDAC3, 5 × 10^8^ TU mL^−1^, forward: 5′‐CCGGCCTGCATTATGGTCTCTATAACTCGAGTTATAGAGACCATAATGCAGGTTTTTG‐3′, reverse: 5′‐AATTCAAAAACCTGCATTATGGTCTCTATAACTCGAGTTATAGAGACCATAATGCAGG‐3′) or the control lentivirus (lenti‐shcon, 5 × 10^8^ TU mL^−1^) was slowly injected into the bilateral hippocampus (anterior–posterior position−2.0 mm, medial–lateral position ± 1.6 mm, dorsoventral 1.5 mm from bregma) of 9‐month‐old APP/PS1 mice via infusion cannulae as previously described (Zhu *et al*., 2014), while the lentivirus overexpressing HDAC3 (lenti‐HDAC3) or the control lentivirus (lenti‐con) was injected into the bilateral hippocampus of 6‐month‐old APP/PS1 mice. The MWM tests were performed 30 days after the lentivirus injection, and the brains were then removed for the other experiments. All animal experiments were approved by the Animal Care Committee of Nanjing University, and every effort was made to minimize the number of experimental animals and their suffering.

### Real‐time PCR

Total RNA was harvested using the TRIzol reagent (Takara, Dalian, Liaoning, China) from the hippocampus of APP/PS1 mice and was reverse‐transcribed into cDNA using a PrimeScript RT reagent kit (Takara). Real‐time PCR was performed using SYBR Fast qPCR Mix (Takara) on an ABI 7500 system (Applied Biosystems, Carlsbad, CA, USA). The mRNA level of HDAC3 was normalized to that of GAPDH. The primers were as follows: HDAC3: forward, TTCAACGTGGGTGATGACTG, reverse, TTAGCTGTGTTGCTCCTTGC; GAPDH: forward, GCCAAGGCTGTGGGCAAGGT, reverse, TCTCCAGGCGGCACGTCAGA.

### Western blot

Nuclear protein was extracted using a Nuclear Extract Kit according to the manufacturer's instructions (Active Motif, Carlsbad, CA, USA). The whole‐cell lysate or nuclear protein was subjected to SDS‐PAGE and transferred onto polyvinylidene difluoride (PVDF) membranes (Millipore, Bedford, MA, USA). The membranes were blocked in 5% nonfat milk for 1 h at room temperature and then incubated overnight at 4°C with the following primary antibodies: HDAC1 (1:1000; Bioworlde, Louis Park, MN, USA), HDAC2 (1:1000; Bioworlde), HDAC3 (1:1000; Bioworlde), HDAC3 (1:1000; Cell Signaling, Danvers, MA, USA), HDAC8 (1:1000; Bioworlde), APP (1:5000; Sigma), ADAM10 (1:1000; Millipore), ADAM17 (1:1000; Millipore), BACE1 (1:1000; Chemicon), Nicastrin (1:1000; Cell Signaling), PEN2 (1:500; Cell Signaling), presenilin 1 (1:1000; Cell Signaling), presenilin 2 (1:500; Cell Signaling), C99 (1:1000; Millipore), Aβ (6E10, 1:1000; Biolegend, San Diego, CA, USA), IDE (1:1000; Abcam), NEP (1:1000; Millipore), LRP1 (1:1000; Abcam, Cambridge, MA, USA), RAGE (1:1000; Abcam), cathepsin B (1:1000; Santa Cruz, Santa Cruz, CA, USA), p‐CREB (1:1000; Cell Signaling), CREB (1:1000; Cell Signaling), synaptophysin (1:1000; Abcam), BDNF (1:1000; Bioworlde), PSD95 (1:1000; Cell Signaling), PSD93 (1:1000; Invitrogen, Carlsbad, CA, USA), GFAP (1:1000; Abcam), Iba‐1 (1:1000; Abcam), AT180 (1:5000; Thermo Fisher), AT270 (1:5000; Thermo Fisher, Lafayette, CO, USA), AT8 (1:5000; Thermo Fisher), AT100 (1:5000; Thermo Fisher), Tau (1:1000; Thermo Fisher), GAPDH (1:5000; Bioworlde), and lamin B (1:1000; Bioworlde). The membranes were washed with TBS‐T and then incubated with secondary antibodies for 1 h at room temperature. The protein signals were detected using an ECL system (Bioworlde), and the band intensities were quantified using imagej software, https://imagej.nih.gov/ij/,NIH, Bethesda, MD, USA .

### Immunofluorescence assay

APP/PS1 mice were anesthetized and perfused with cold saline, followed by 4% paraformaldehyde (PFA). The brain sections were blocked with 3% BSA for 1 h and incubated overnight with primary antibodies: Iba‐1 (1:400; Abcam), GFAP (1:400; BD), and Aβ (1:200; Biolegend) at 4°C, followed by incubation with the secondary antibodies for 1 h at room temperature. The nuclei were stained with DAPI reagent (Bioworlde). Qualitative immunostaining analyses were conducted as previously described (Zhao *et al*., 2013). The immunoreactive cells to GFAP and Iba‐1 in about 100 areas of 1 × 1 mm in five slices per brain were counted. The Aβ plaque load was determined by imagej software and expressed as the percent of total brain area for each slice. All procedures were performed in a randomized and blinded manner.

### Aβ ELISA

Soluble and insoluble Aβ in the brains were sequentially extracted as described elsewhere (Youmans *et al*., 2011). In brief, brains were homogenized in 15 volumes (w/v) of TBS buffer with phosphatase and protease inhibitor cocktails (Sigma) and then centrifuged at 100 000 *g* for 1 h at 4°C. The supernatant was collected as the TBS‐soluble fraction (soluble Aβ). The pellet was resuspended in 15 volumes of 1% Triton X‐100/TBS (TBS‐X), incubated on ice for 30 min and then centrifuged at 100 000 *g* for 1 h at 4°C. The supernatant was removed as the TBS‐X‐soluble fraction. The pellet was resuspended in 70% formic acid (FA) and centrifuged at 100 000 *g* for 1 h at 4°C. The supernatant was neutralized with 1 M Tris base (pH 11), representing the FA‐soluble fraction (insoluble Aβ). The protein levels of the TBS‐soluble and TBS‐X‐soluble fractions were quantified using a BCA protein assay kit (Bioworlde), and the protein levels of the FA‐soluble fractions were detected using a Bradford protein assay kit (Bioworlde). The levels of Aβ_1–40_ and Aβ_1–42_ in each fraction were quantified using Quantikine ELISA Human Amyloid β aa1‐40/aa1‐42 Immunoassay kits (R&D Systems, Minneapolis, MN, USA) following the manufacturer's instructions.

### Golgi staining and dendritic spine analysis

Golgi staining was performed using an FD Rapid GolgiStain Kit (FD Neurotechnologies, Columbia, MD, USA) as previously described (Wang *et al*., 2016). Briefly, brain tissues were immersed in impregnation solution consisting equal volumes of Solutions A and B for 2 weeks and then removed to Solution C for 3 days at room temperature in the dark. Brain sections (200 μm thickness) were placed on Solution C‐coated microscope slides and transferred into in a mixture including DDW, Solution D and Solution E for 10 min, then rinsed in DDW, dehydrated, dried, cleared and covered with coverslips. The sections were observed using an OLYMPUS BX51 microscope, and the pyramidal neurons in the CA1 region of the hippocampus were analyzed. For each mouse, five neurons were randomly selected, and three segments (at least 30 μm) were randomly chosen per neuron from the apical and basal dendrites, and the numbers of spines per 10 μm were counted in a blinded manner using imagej software.

### Morris water maze (MWM) tests

The MWM tests were performed as previously reported (Zhu *et al*., 2014). Briefly, during the acquisition trial (days 1–5), each mouse was trained to find a submerged platform within 60 s, and the latency, swimming speed and searching distance were recorded using any‐maze software (Stoelting, USA). If the mouse found the platform within 60 s, the time was recorded as the latency. Otherwise, the mouse was gently guided to the platform and allowed to remain on the platform for 10 s, and the latency was recorded as 60 s. For the probe trial (day 6), the platform was removed and the mouse was allowed to swim freely for 60 s; the number of target platform crossings and the time spent and swimming distances in each quadrant were recorded.

### Statistical analysis

All data were expressed as mean ± standard error of mean (SEM) and analyzed using spss 18.0. Differences in escape latency, searching distance and swimming speed in the MWM tests were analyzed using two‐way analysis of variance (ANOVA), and all other data were analyzed using Student's *t*‐test. *P *<* *0.05 was considered statistically significant.

## Funding

This work was supported by the National Natural Science Foundation of China (81200839 and 81671055 to XLZ, 81300988 to JLJ, and 81230026, 81630028, and 81171085 to YX), and Jiangsu Provinical Key Medical Discipline (ZDXKA2016020 to YX).

## Author contributions

YX conceived and designed the research; XLZ, SLW, LJY, and JLJ carried out experiments; XY and YL analyzed the data; and XLZ, SLW, and YX wrote the manuscript.

## Conflict of interest

The authors declare no conflict of interest.

## Supporting information


**Fig. S1** The mRNA levels of HDAC3 in the hippocampus of 6‐ and 9‐month‐old APP/PS1 mice were not significantly changed.

**Fig. S2** Lentivirus‐medicated overexpression of HDAC3 in the hippocampus increases the escape latency and searching distance of 6‐month‐old APP/PS1 mice in the MWZ tests.

**Fig. S3** HDAC3 overexpression increases Aβ levels in the hippocampus of 6‐month‐old APP/PS1 mice.

**Fig. S4** HDAC3 overexpression increases the level of PS1 and decreases the level of ADAM10 in the hippocampus of 6‐month‐old APP/PS1 mice.

**Fig. S5** HDAC3 overexpression reduces dendritic spine density in the hippocampus of APP/PS1 mice.

**Fig. S6** HDAC3 overexpression exacerbates microglial activation in the hippocampus of APP/PS1 mice.

**Fig. S7** HDAC3 does not affect Tau phosphorylation in the hippocampus of APP/PS1 mice.
