# Supplementary figures and images for: HDAC3 negatively regulates spatial memory in a mouse model of Alzheimer's disease
Source: Aging Cell. 2017 Aug 3;16(5):1073–82. doi: 10.1111/acel.12642 (PMC5595690; doi:10.1111/acel.12642)

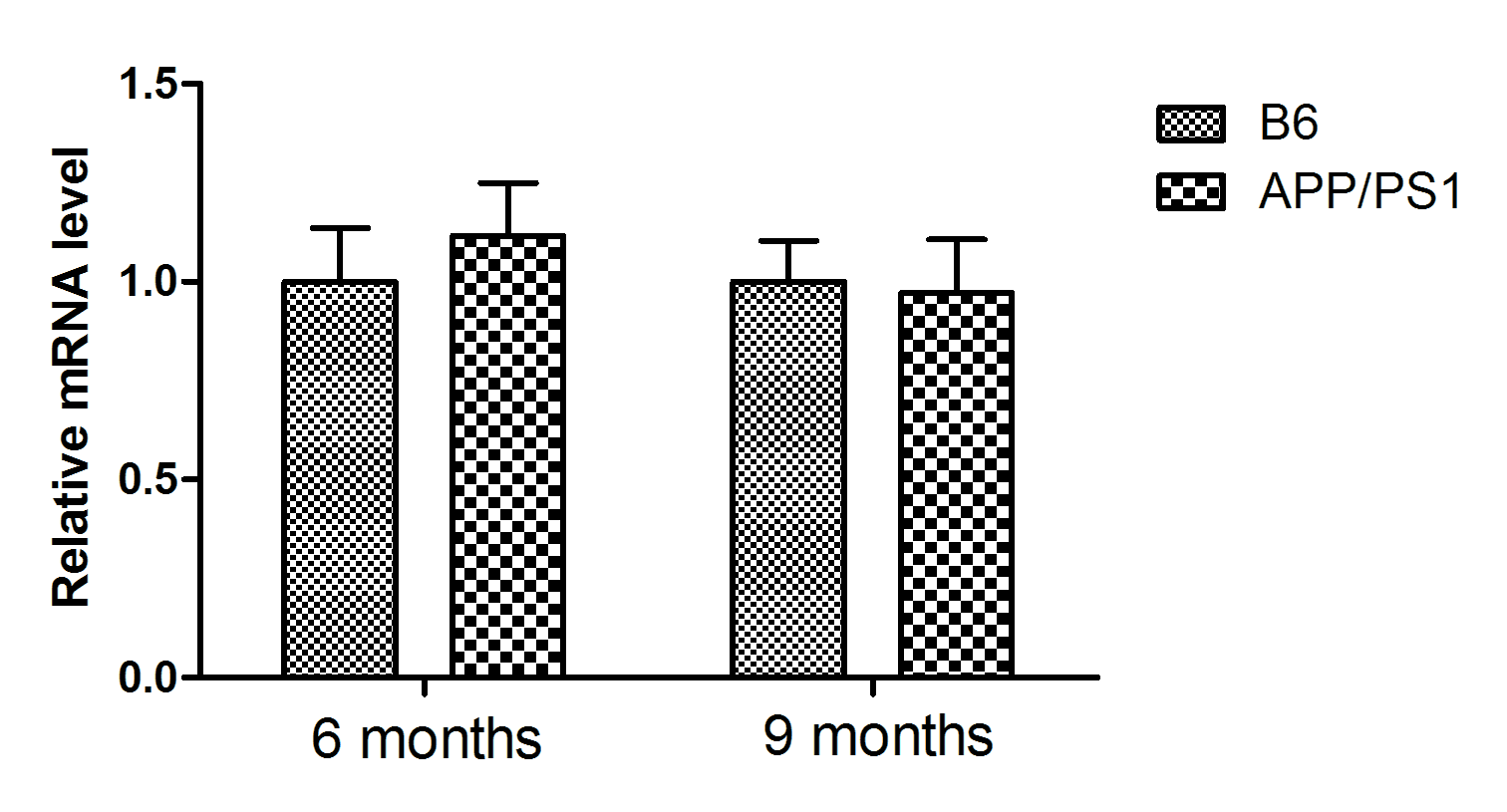

Supplement: Supplementary file 1 — Fig. S1 The mRNA levels of HDAC3 in the hippocampus of 6‐ and 9‐month‐old APP/PS1 mice were not significantly changed. [file ACEL-16-1073-s001.tif]

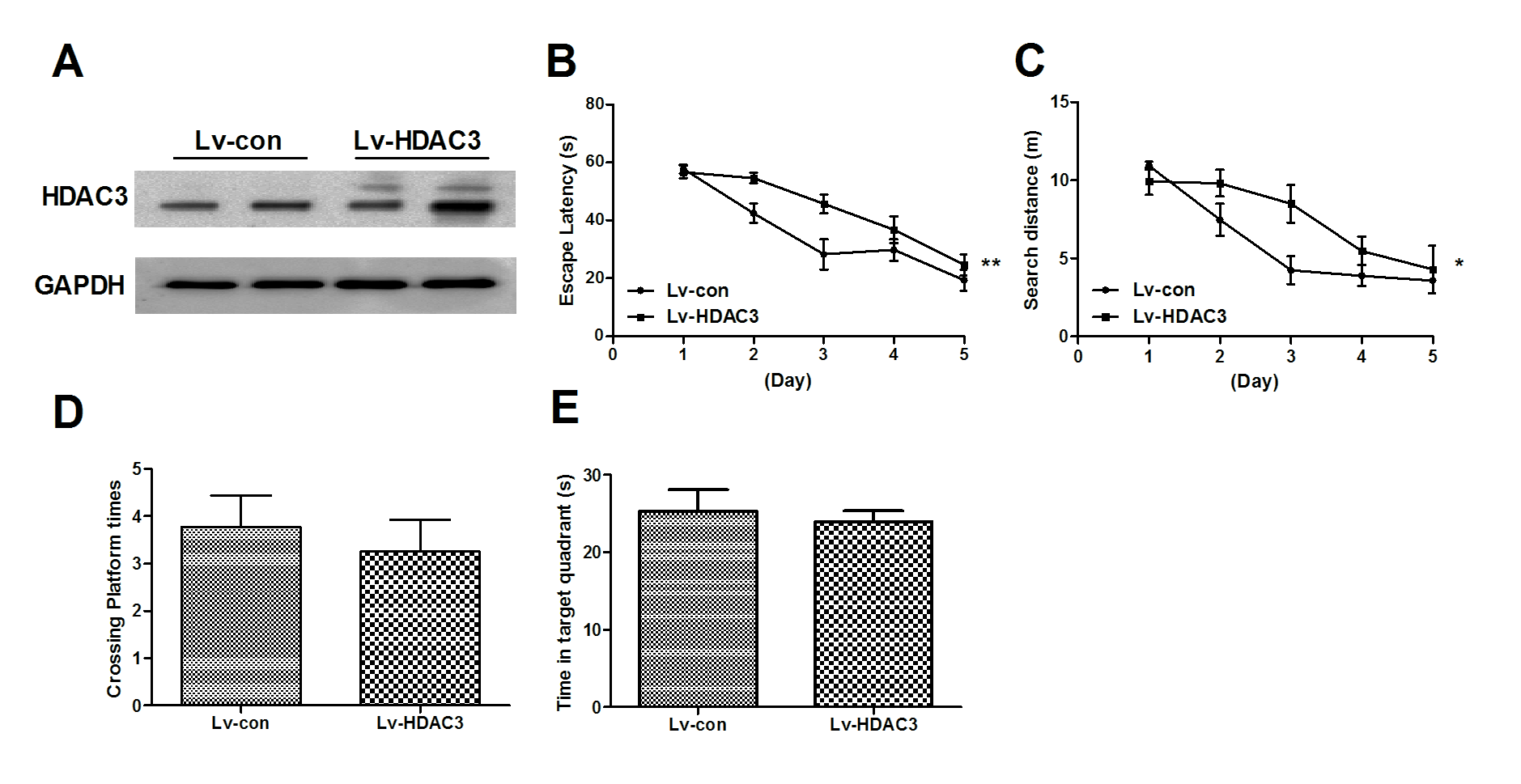

Supplement: Supplementary file 2 — Fig. S2 Lentivirus‐medicated overexpression of HDAC3 in the hippocampus increases the escape latency and searching distance of 6‐month‐old APP/PS1 mice in the MWZ tests. [file ACEL-16-1073-s002.tif]

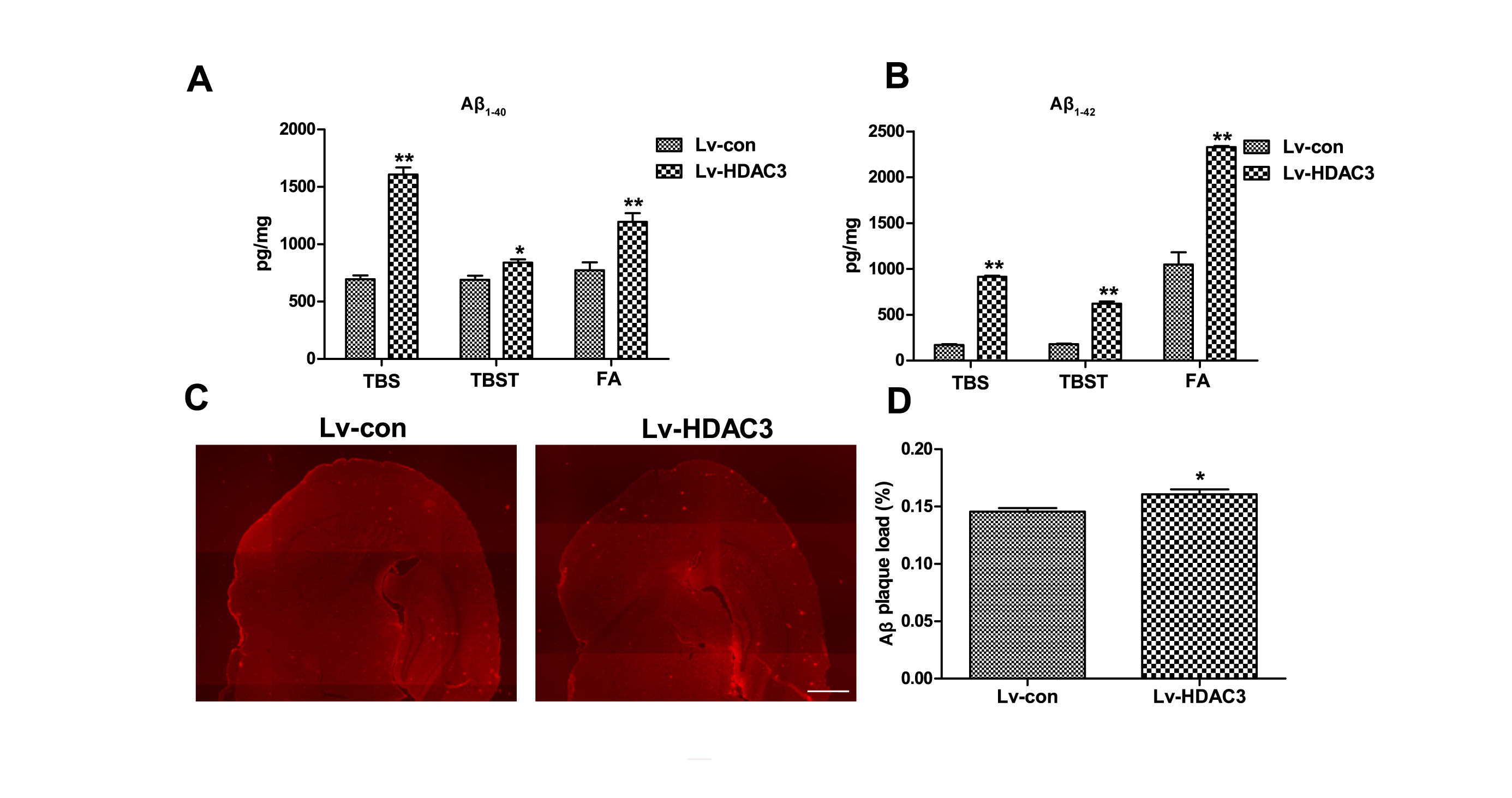

Supplement: Supplementary file 3 — Fig. S3 HDAC3 overexpression increases Aβ levels in the hippocampus of 6‐month‐old APP/PS1 mice. [file ACEL-16-1073-s003.tif]

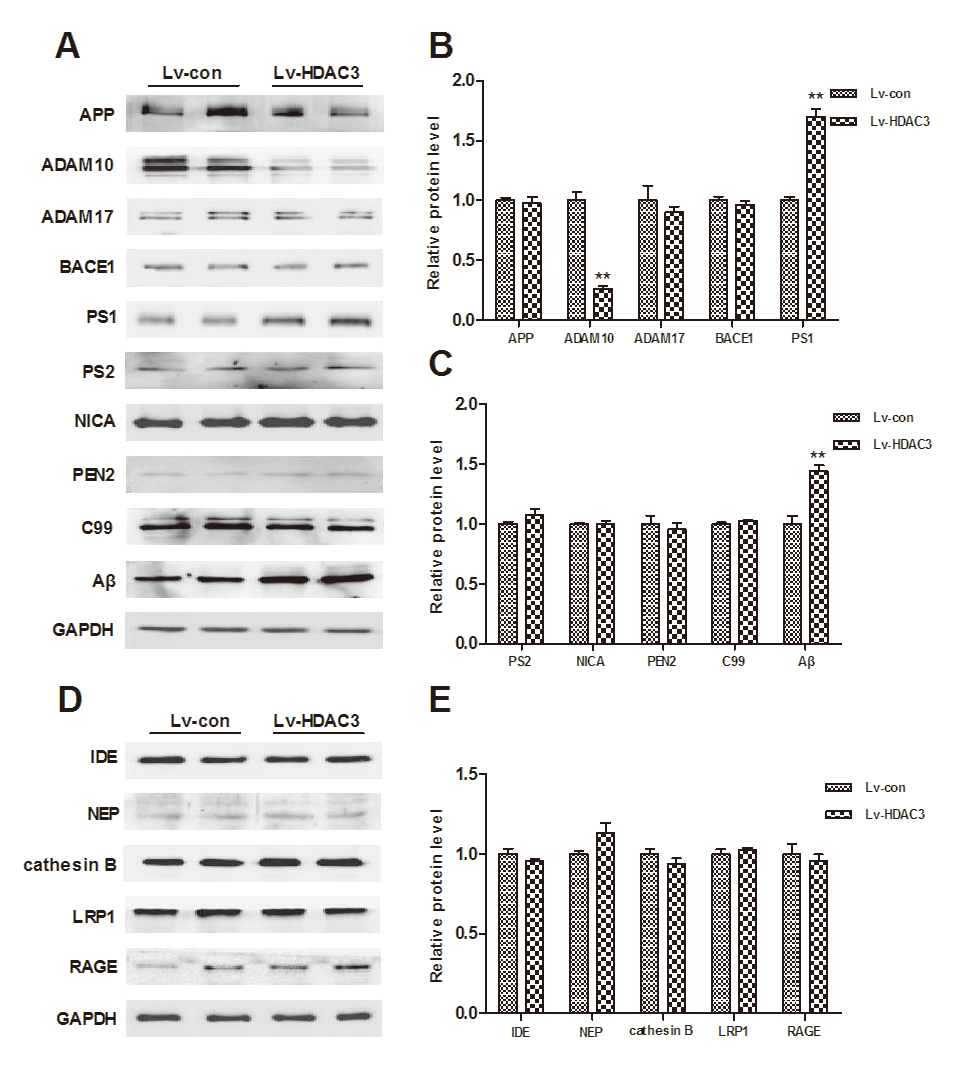

Supplement: Supplementary file 4 — Fig. S4 HDAC3 overexpression increases the level of PS1 and decreases the level of ADAM10 in the hippocampus of 6‐month‐old APP/PS1 mice. [file ACEL-16-1073-s004.tif]

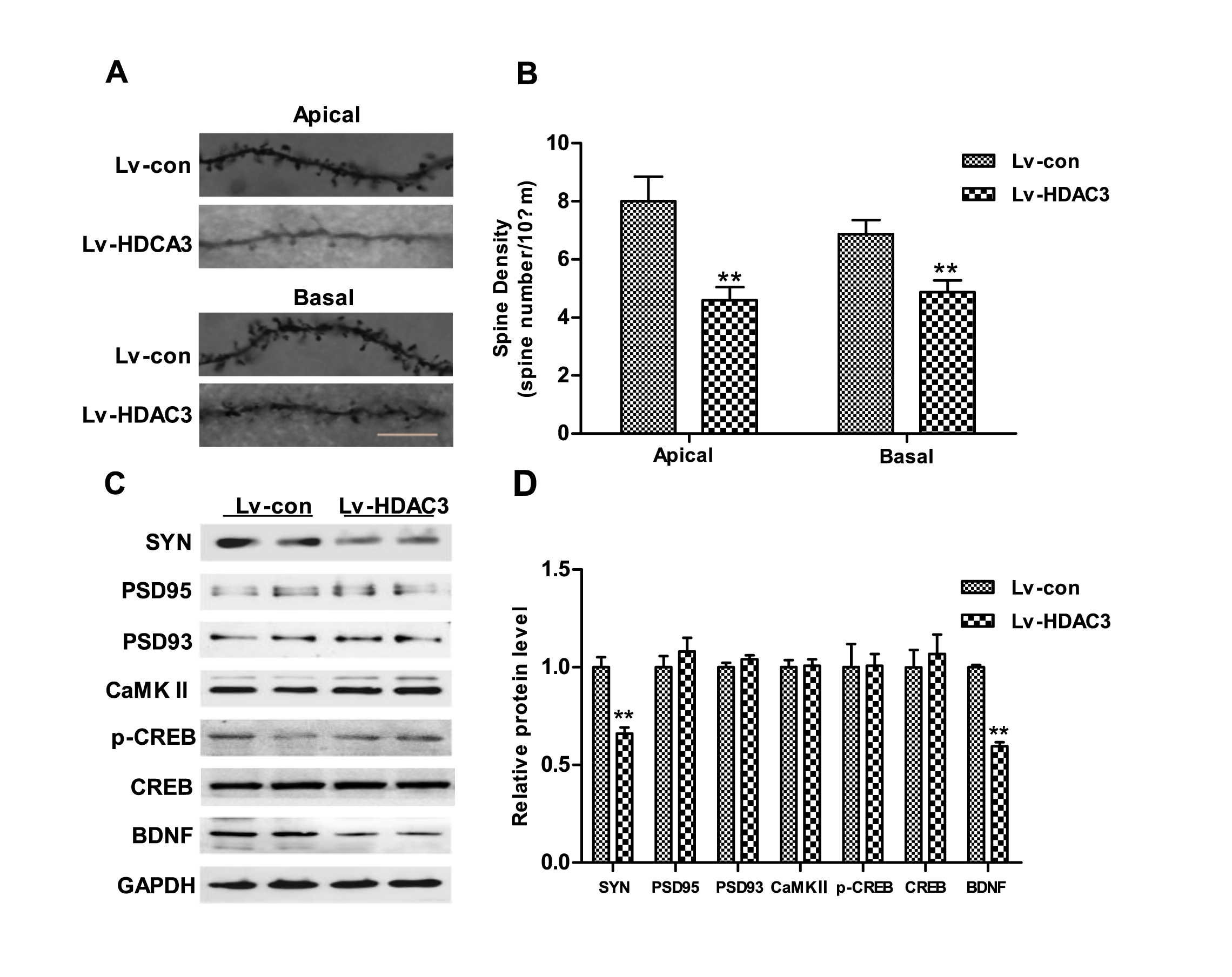

Supplement: Supplementary file 5 — Fig. S5 HDAC3 overexpression reduces dendritic spine density in the hippocampus of APP/PS1 mice. [file ACEL-16-1073-s005.tif]

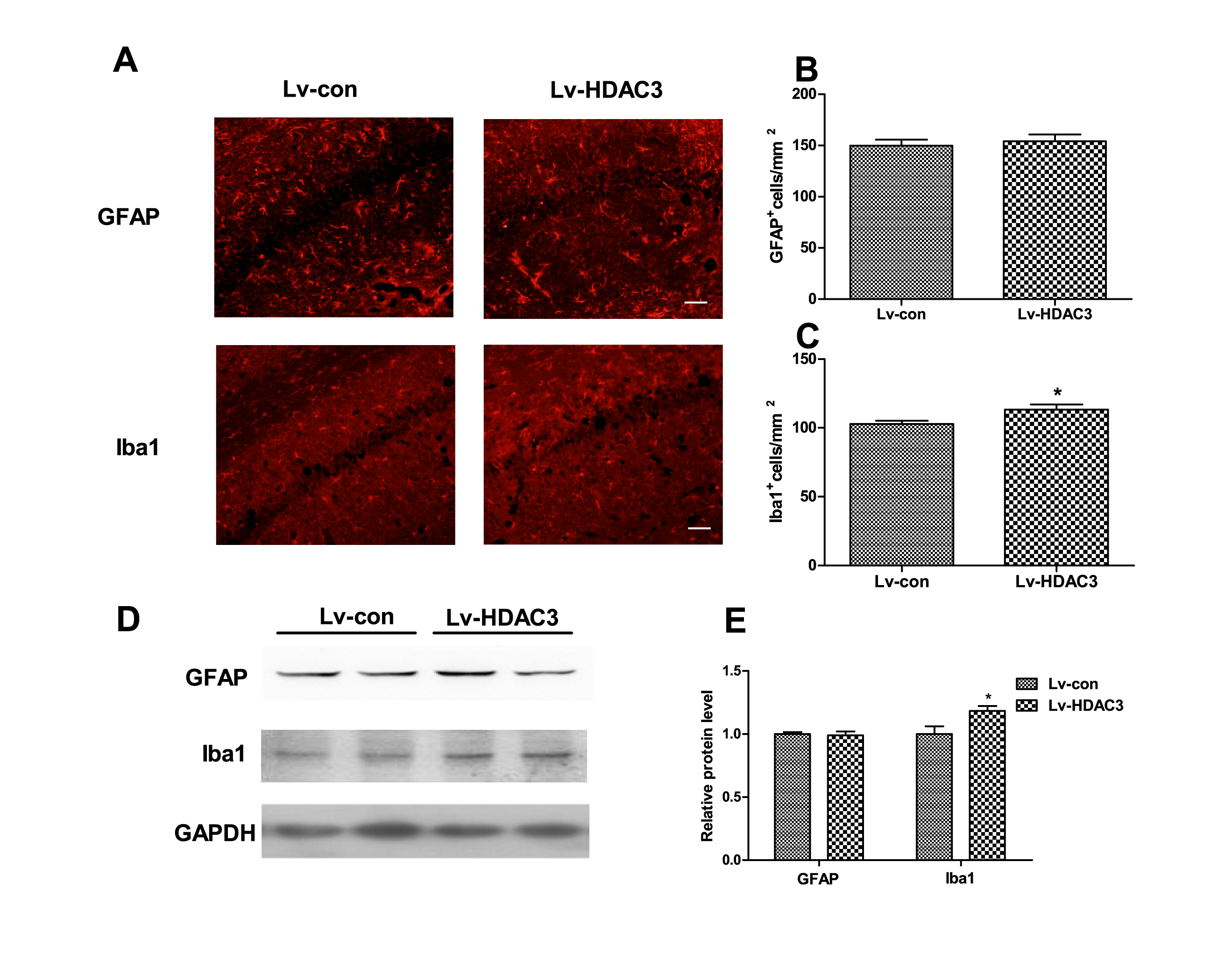

Supplement: Supplementary file 6 — Fig. S6 HDAC3 overexpression exacerbates microglial activation in the hippocampus of APP/PS1 mice. [file ACEL-16-1073-s006.tif]

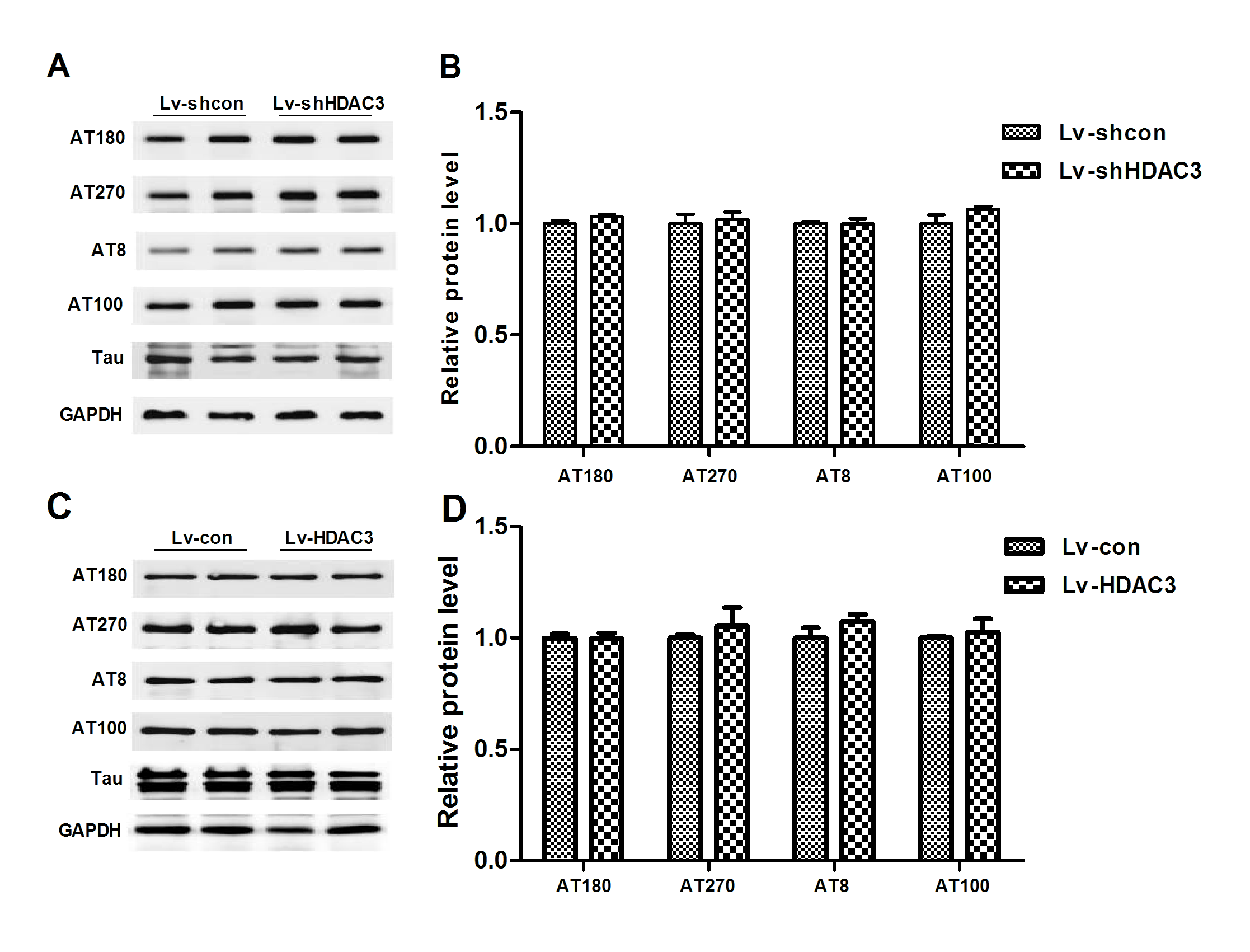

Supplement: Supplementary file 7 — Fig. S7 HDAC3 does not affect Tau phosphorylation in the hippocampus of APP/PS1 mice. [file ACEL-16-1073-s007.tif]
