# Supplementary material for: HDAC3 negatively regulates spatial memory in a mouse model of Alzheimer's disease
Source: Aging Cell. 2017 Aug 3;16(5):1073–82. doi: 10.1111/acel.12642 (PMC5595690; doi:10.1111/acel.12642)
Supplement: Supplementary file 8 [file ACEL-16-1073-s008.doc]

**Supplementary Figure Legends**

**HDAC3 negatively regulates spatial memory in a mouse model of Alzheimer’s disease**

**Xiaolei Zhu,^1,2,3, *^ Sulei Wang,^4, *^ Linjie Yu,^1^ Jiali Jin,^1^ Xing Ye,^1^ Yi Liu,^1^ Yun Xu^1,2,3^**

^1^ Department of Neurology, Drum Tower Hospital, Medical School and The State Key Laboratory of Pharmaceutical Biotechnology, Nanjing University, Nanjing, PR China

^2^ Jiangsu Key Laboratory for Molecular Medicine, Medical School of Nanjing University, Nanjing, PR China

^3^ Nanjing Neuropsychiatry Clinic Medical Center, Nanjing, PR China

^4^ Department of Neurology, Nanjing Hospital of Traditional Chinese Medicine, Nanjing, PR China

Correspondence to: Yun Xu, Department of Neurology, Drum Tower Hospital, Medical School and The State Key Laboratory of Pharmaceutical Biotechnology, Nanjing University, Zhongshan Road321#, Nanjing, Jiangsu Province, 210008, PR China. E-mail: xuyun20042001@aliyun.com.

*These authors contributed equally to this study.

**Running title: HDAC3 negatively regulates spatial memory**

**Fig. S1** The mRNA levels of HDAC3 in the hippocampus of 6- and 9-month-old APP/PS1 mice were not significantly changed. The mRNA levels of HDAC3 was determined by real-time PCR. n=6 mice per group.

**Fig. S2** Lentivirus-medicated overexpression of HDAC3 in the hippocampus increases the escape latency and searching distance of 6-month-old APP/PS1 mice in the MWZ tests. Six-month-old APP/PS1 mice were injected with lenti-HDAC3 or lenti-con, and the MWM tests were performed 30 days later. (A) The level of HDAC3 in the hippocampus was determined by western blot after lenti-HDAC3 injection. n=4 mice per group. In the acquisition trial, the escape latency (B) and searching distance (C) were recorded in lenti-HDAC3 (n=8) and lenti-con (n=6) injected mice, and the time of target platform crossings (D, *p*= 0.586, ns) and time in the target quadrant (F, *p*= 0.664, ns) were examined in the probe trials. * *p*< 0.05, ** *p*< 0.01.

**Fig. S3** HDAC3 overexpression increases Aβ levels in the hippocampus of 6-month-old APP/PS1 mice. (A) The levels of TBS-, TBS-X-, and FA-soluble Aβ_1-40_ in the hippocampus were determined by ELISA. n=4-6 mice per group. (B) The levels of TBS-, TBS-X-, and FA-soluble Aβ_1-42_ in the hippocampus were determined by ELISA. n=4-6 mice per group. (C) Representative image of Aβ staining (6E10) in the brains of lenti-HDAC3-injected APP/PS1 mice. bar=1 mm. n=3-4 mice per group. (D) The area percentage of 6E10-positive Aβ plaque load in the brains. * *p*< 0.05, ** *p*< 0.01.

**Fig. S4** HDAC3 overexpression increases the level of PS1 and decreases the level of ADAM10 in the hippocampus of 6-month-old APP/PS1 mice. (A) The levels of APP and secretases of lenti-HDAC3 injected APP/PS1 mice were examined by western blot. n=4-5 mice per group. (B, C) Graph represented quantification of the signal intensities normalized to GAPDH as a loading control. (D) The levels of Aβ-metabolism-associated enzymes were determined in the hippocampus of lenti-HDAC3 injected APP/PS1 mice. n=4-5 mice per group. (E) Graph represented quantification of the signal intensities normalized to GAPDH as a loading control. ** *p<*0.01.

**Fig. S5** HDAC3 overexpression reduces dendritic spine density in the hippocampus of APP/PS1 mice. (A) Representative image of Golgi-stained apical and basal dendritic branches. bar = 10 μm. n=3-4 mice per group. (B) Graph represented quantification of the dendritic spine density. (C) The levels of synaptophysin, PSD95, PSD93, CaMKII, p-CREB, CREB and BDNF of lenti-HDAC3 injected APP/PS1 mice were determined by western blot. n=4-5 mice per group. (D) Graph represented quantification of the signal intensities normalized to GAPDH as a loading control. ** *p*< 0.01.

**Fig. S6** HDAC3 overexpression exacerbates microglial activation in the hippocampus of APP/PS1 mice. (A) Representative image of astrocytes and microglia stained with GFAP and Iba-1, respectively, in the hippocampus of lenti-HDAC3-injected APP/PS1 mice. bar=50 μm. n=4-5 mice per group. (B, C) Graph represented quantification of the signal intensities. (D) The levels of GFAP and Iba-1 in the hippocampus were determined by western blot. n=4-6 mice per group. (E) Graph represented quantification of the signal intensities normalized to GAPDH as a loading control. * *p*< 0.05.

**Fig. S7** HDAC3 does not affect Tau phosphorylation in the hippocampus of APP/PS1 mice. (A) Tau phosphorylation was determined by western blot in the hippocampus of Lv-shHDAC3-injected APP/PS1 mice. n=4-6 mice per group. (B) Graph represented quantification of the signal intensities normalized to Tau as a control. (C) Tau phosphorylation was determined by determined by western blot in the hippocampus of Lv-HDAC3-injected APP/PS1 mice. n=4-6 mice per group. (D) Graph represented quantification of the signal intensities normalized to Tau as a control.
